# Intelligent fault detection strategy for knowledge entities in fault semantic networks of distribution network based on siamese networks

**DOI:** 10.1371/journal.pone.0303084

**Published:** 2024-05-16

**Authors:** Xinjie Sun, Tao Qin, Lingyun Tong, Haoliang Zhang, Weihan Xu

**Affiliations:** State Grid Nanjing Power Supply Company, Nanjing, China; Helwan University Faculty of Engineering, EGYPT

## Abstract

The advent of smart grid technologies has brought about a paradigm shift in the management and operation of distribution networks, allowing for intricate system information to be encapsulated within semantic network models. These models, while robust, are not immune to faults within their knowledge entities, which can arise from a myriad of issues, potentially leading to verification failures and operational disruptions. Addressing this critical vulnerability, our research delves into the development of a novel fault detection methodology specifically tailored for the knowledge entity variables of semantic networks in distribution networks. In our approach, we first construct a state space equation that models the behavior of knowledge entity variables in the presence of faults. This foundational framework enables us to apply an unknown input observer strategy to effectively detect anomalies within the system. To bolster the fault identification process, we introduce the innovative use of a siamese network, a neural network architecture which is proficient in differentiating between similar datasets. Through simulation scenarios, we demonstrate the efficacy of our proposed fault detection method.

## Introduction

The inception of smart grid technology has precipitated a paradigm shift in the domain of power distribution networks, heralding unprecedented levels of operational efficiency, enhanced reliability, and a harmonious integration of renewable energy sources [1–3]. As conduits between electricity suppliers and end-users, distribution networks have evolved into intricate and dynamic systems [4]. This evolution has necessitated the deployment of sophisticated information and communication technologies to adeptly manage the burgeoning data landscape, ensuring fluid grid operations [5, 6].

Within this advanced operational context, semantic network models have risen to prominence, providing an essential framework for the representation of system information. These models are instrumental in elucidating and orchestrating the complex interplay among the diverse components of the smart grid ecosystem [7, 8].

Central to these semantic networks are the knowledge entities, which serve as proxies for the system’s components and their elaborate interconnections [9]. These entities are the linchpin in the accurate representation of distribution network behavior, embodying critical data pertaining to the operational states of tangible infrastructure elements, including transformers, power lines, and load-bearing units. The fidelity of these knowledge entities is non-negotiable, as any divergence from their true state can trigger a cascade of operational inefficiencies, misguided decision-making, or in the worst cases, outright system failures [10]. Thus, the reliability of distribution networks is inextricably linked to the precision and steadfastness of the data held within these knowledge entities [11].

Despite their foundational role, knowledge entities are not impervious to faults, which can emanate from a variety of sources such as sensor dysfunctions, communication breakdowns, or anomalies in data processing [12–14]. These faults pose a significant threat, potentially undermining the verification processes and jeopardizing the operational integrity of the distribution network [15–17].

Confronting these challenges is of paramount importance, necessitating focused research efforts towards the development of intelligent fault detection methodologies that are specifically designed for the nuanced requirements of knowledge entities in semantic networks [18, 19]. In this paper, we introduce a cutting-edge fault detection approach for knowledge entity variables within distribution networks. This approach is underpinned by a state space equation that anticipates potential faults, coupled with the implementation of an unknown input observer adept at discerning system states amidst the presence of unmeasured disturbances.

To augment the fault detection capabilities, we incorporate Siamese networks into our methodology. These neural networks are renowned for their proficiency in similarity detection and pattern recognition, which significantly bolsters our ability to identify and rectify dual source data discrepancies within knowledge entities. Through this research, we aim to not only underscore the criticality of maintaining knowledge entity integrity but also deliver a robust and reliable framework to protect the smart grid infrastructure from the adverse effects of data faults.

The contributions of this paper are multifaceted and represent a significant advancement in the field of smart grid management.

1. We develope a state space equation tailored to the state variables of knowledge entities, explicitly taking into account the potential fault scenarios that may afflict distribution networks. This formulation provides a comprehensive description of the system’s operational state, reflecting a deep understanding of the dynamics at play within the smart grid.
2. We employ an unknown input observer, a sophisticated tool designed to discern fault information within the distribution network. This approach leverages the observer’s inherent capability to detect anomalies, thereby enhancing the reliability of the fault diagnosis process.
3. We apply Siamese networks, leveraging their exceptional pattern recognition abilities, to identify and isolate fault information in the distribution network. This application showcases the potential of advanced neural networks in improving fault detection mechanisms within complex systems.

## Modeling of state space for knowledge entities considering the fault scenarios

The operational status methodology for the distribution network that this article presents meticulously incorporates considerations of power shortage and scheduling costs [20, 21]. Within this framework, power-related state space variables serve to estimate the magnitude of power shortages afflicting the distribution network, while cost-related state space variables are instrumental in calculating the incremental costs associated with the optimal scheduling solutions for the distribution network’s challenges.

It is critical to acknowledge that the integrity of knowledge entity information within the semantic network is foundational to the precision of our estimations. Any deviation in this data directly impacts the accuracy of power shortage assessments and the computation of incremental costs, which, in turn, bears upon the stability and economic viability of the distribution network’s operations.

The operational state space equation that accounts for power shortages and scheduling costs within a distribution network is formulated as follows:

$$\begin{array}{c} \left\{ \begin{array}{c} x(k+1)=Ax(k)+Bu(k),\;normal\\ x(k+1)=Ax(k)+Bu(k)+Mm(k),\;compromised \end{array}\right. \end{array}$$ (1)

where

$$\begin{array}{l} x={\left[x_{p}^{P}\;x_{p}^{C}\;x_{h}^{C}\;x_{h}^{H}\right]}^{T}\\ u={\left[u_{p}^{P}\;u_{p}^{C}\;u_{h}^{C}\;u_{h}^{H}\right]}^{T} \end{array}$$

where $x_{p}^{P}$ and $x_{p}^{C}$ respectively represent the fault handling capabilities of traditional and distributed resources in the distribution network; $x_{h}^{C}$ and $x_{h}^{H}$ represent the fault handling costs of traditional and distributed resources in the distribution network, respectively; $u_{p}^{P}$ and $u_{p}^{C}$ respectively represent the deviation between the fault handling capabilities of traditional and distributed resources in the distribution network and the expected ones; $u_{h}^{C}$ and $u_{h}^{H}$ respectively represent the deviation between the fault handling cost of traditional and distributed resources in the distribution network and the expected cost; *A* and *B* are knowledge graph matrices for fault handling in distribution networks; *m*(*k*) and *M* are the matrixes to represent the fault.

It can be understood that changes in the state variables of knowledge entities in different stages of the distribution network fault model will affect the effectiveness of distribution network fault handling. In the next section, observers with different fault knowledge entity variables were designed to observe faults in the distribution network.

## Design of observers for detecting compromised variables in knowledge graph of distribution network

### Observer design of fault handling capabilities considering faults

In this subsection, we focus on the observer for fault handling capabilities. The original system Σ_*fhc*_ is

$$\begin{array}{c} \left\{ \begin{array}{c} x(k+1)=Ax(k)+Bu(k)+Mm(k)\\ y(k)=Cx(k) \end{array}\right. \end{array}$$ (2)

By incorporating the fault vector *m*(*k* − 1) from time *k* − 1 as an additional state, we enhance our analytical capabilities. This incorporation leads to the formulation of an augmented state vector $\bar{x}\left(k\right)={\left[x\left(k\right)\;m\left(k-1\right)\right]}^{T}$. With this enriched state representation, we are able to construct an augmented system that provides a more comprehensive understanding of the network’s dynamics in the presence of faults. This augmented system is presented as follows:

$$\begin{array}{c} \left\{ \begin{array}{c} \bar{E}\bar{x}\left(k+1\right)=\bar{A}\bar{x}\left(k\right)+\bar{B}u\left(k\right)\\ y\left(k\right)=\bar{C}\bar{x}\left(k\right) \end{array}\right. \end{array}$$ (3)

where

$$\begin{array}{c} \bar{E}=[\begin{array}{cc} I_{n} & -M\\ 0 & 0 \end{array}],\bar{A}=[\begin{array}{cc} A & 0\\ 0 & 0 \end{array}],\bar{B}=[\begin{array}{c} B\\ 0 \end{array}],\bar{C}={\left[\begin{array}{c} C\\ 0 \end{array}\right]}^{T} \end{array}$$

The observer for the augmented system is delineated as follows:

$$\begin{array}{c} \left\{ \begin{array}{cc} z\left(k+1\right) & =R\bar{A}\hat{\bar{x}}\left(k\right)+R\bar{B}u\left(k\right)+L\left(y\left(k\right)-\bar{C}\hat{\bar{x}}\left(k\right)\right)\\ \hat{\bar{x}}\left(k\right) & =z\left(k\right)+Ty\left(k\right) \end{array}\right. \end{array}$$ (4)

Within this framework, let *z* represent the dynamics of system (3); $\hat{\bar{x}}\left(k\right)$ signifies the estimate of the augmented state vector $\bar{x}\left(k\right)$. To optimize the observer’s performance, we introduce gain matrices *R*, *L* and *T* each carefully dimensioned to align with the system’s parameters. These matrices are not arbitrary; they are the result of a rigorous design process aimed at achieving optimal state estimation fidelity.

**Theorem 1.** The state observer, as delineated in form (4), must adhere to a set of precisely defined criteria to ensure its effectiveness and reliability. These requirements are twofold: 1)The first condition necessitates an algebraic relationship where the matrix *R*, when multiplied by the augmented system matrix $\bar{E}$, and the matrix *T*, when multiplied by the augmented output matrix $\bar{C}$, must collectively yield the identity matrix *I*_*n*+*q*_;. This identity matrix has dimensions that correspond to the sum of the system’s state dimensions and the fault vector’s dimensions, ensuring that the observer gain matrices are correctly calibrated for accurate state reconstruction. 2)The second condition requires the existence of symmetric positive definite matrices *P* and *W*. These matrices are not merely mathematical constructs; they are pivotal in defining a Lyapunov function that guarantees the convergence and stability of the observer. The satisfaction of these conditions is essential for the observer to perform its function with the requisite level of precision and robustness. The precise mathematical formulations that codify these requirements are as follows:

$$\begin{array}{c} {}[\begin{array}{cc} -P & {\left(R\bar{A}\right)}^{T}P-{\bar{C}}^{T}W^{T}\\ {}* & -P \end{array}]<0 \end{array}$$ (5)

**proof.** We have the following matrices $U\in R^{\left(n+q)\times (n+q\right)}$ and $V\in R^{\left(n+q)\times (n+q\right)}$ satisfied

$$\begin{array}{c} U\bar{E}V=[\begin{array}{cc} I_{N} & 0\\ 0 & 0 \end{array}] \end{array}$$ (6)

So that

$$\begin{array}{cc} rank[\begin{array}{cc} I_{n} & 0\\ 0 & 0\\ C & 0 \end{array}] & =rank\{[\begin{array}{cc} U & 0\\ 0 & I_{m} \end{array}][\begin{array}{c} \bar{E}\\ \bar{C} \end{array}]V\}\\ & =rank[\begin{array}{c} \bar{E}\\ \bar{C} \end{array}]=n+q \end{array}$$ (7)

Therefore

$$\begin{array}{c} rank[\begin{array}{cc} I_{N} & 0\\ C & 0 \end{array}]=rank[\begin{array}{c} \bar{E}\\ \bar{C} \end{array}]=n+q \end{array}$$ (8)

$$\begin{array}{c} rank\left(U\bar{E}V+\bar{C}V\right)=rank[\begin{array}{cc} I_{N} & 0\\ C & 0 \end{array}]=n+q \end{array}$$ (9)

*R* satisfied

$$\begin{array}{c} R=V{\left(U\bar{E}V+CV\right)}^{-1}U \end{array}$$ (10)

*T* satisfied

$$\begin{array}{c} T=V{\left(U\bar{E}V+CV\right)}^{-1} \end{array}$$ (11)

So, $R\bar{E}+T\bar{C}=I_{n+q}$. [*R*, *T*] and ${\left[\bar{E}\bar{C}\right]}^{T}$ satisfied

$$\begin{array}{c} \left[\begin{array}{c} \bar{E}\\ \bar{C} \end{array}][\begin{array}{cc} R & T \end{array}][\begin{array}{c} \bar{E}\\ \bar{C} \end{array}]=[\begin{array}{c} \bar{E}\\ \bar{C} \end{array}\right] \end{array}$$ (12)

Therefore, we can derive

$$\begin{array}{c} \left[\begin{array}{cc} R & T \end{array}]={\left[\begin{array}{c} \bar{E}\\ \bar{C} \end{array}\right]}^{†}+\Theta (I_{n+q+m}-[\begin{array}{c} \bar{E}\\ \bar{C} \end{array}]{\left[\begin{array}{c} \bar{E}\\ \bar{C} \end{array}\right]}^{†}\right) \end{array}$$ (13)

The matrix Θ, which resides in the real-valued space $R^{\left(n+q)\times (n+q+m\right)}$ is not arbitrarily chosen. rather, it is selected with a deliberate intention that is grounded in the fundamental principles of system design.

As to the *e*(*k*), we have

$$\begin{array}{c} e\left(k\right)=\bar{x}\left(k\right)-\bar{\dot{x}}\left(k\right) \end{array}$$ (14)

Therefore

$$\begin{array}{cc} e(k+1)= & \left(R\bar{E}+T\bar{C}\right)x\left(k+1\right)-z\left(k+1\right)-Ty\left(k+1\right)\\ & =R\bar{E}x\left(k+1\right)-z\left(k+1\right)\\ & =\left(R\bar{A}-L\bar{C}\right)e\left(k\right) \end{array}$$ (15)

Since

$$\begin{array}{c} V\left(k\right)=e^{T}\left(k\right)Pe\left(k\right),\;\;P>0 \end{array}$$ (16)

We have

$$\begin{array}{cc} \Delta V(k) & =V(k+1)-V(k)\\ & =e^{T}\left(k\right)\left[{\left(R\bar{A}-L\bar{C}\right)}^{T}P\left(R\bar{A}-L\bar{C}\right)-P\right]e\left(k\right) \end{array}$$ (17)

If the following equation holds

$$\begin{array}{c} {}[\begin{array}{cc} -P & {\left(R\bar{A}-L\bar{C}\right)}^{T}P\\ P(R\bar{A}-L\bar{C}) & -P \end{array}]<0 \end{array}$$ (18)

By invoking the Schur complement theorem in conjunction with the principles of Lyapunov stability theory, we rigorously establish that Δ*V*(*k*) < 0. This result is not merely a theoretical assertion but a demonstrable guarantee of the system’s performance. It signifies that the Lyapunov function *V*(*k*) is strictly decreasing, thereby providing a robust mathematical argument for the convergence of the error term *e*(*k*).

The completion of the proof provides a substantive insight into the resilience of the proposed observer within our control system. Specifically, it has been demonstrated that, even in the event of a compromise to the incremental cost estimator, the defender retains the capability to accurately monitor system variables. This resilience is not an incidental feature but a deliberate design element, ensuring that the integrity of the system’s observational mechanisms is preserved under adverse conditions. The robustness of the proposed observer is thus affirmed, underscoring its potential as a reliable tool for maintaining situational awareness and operational continuity within the system.

### Observer design of fault handling costs calculation under faults

In this subsection, we focus on the observer for compromised fault handling costs calculation. The system Σ_*fhcc*_ can be expressed as

$$\begin{array}{c} \left\{ \begin{array}{c} x(k+1)=Ax(k)+Bu(k)\\ y(k)=Cx(k)+Nm(k) \end{array}\right. \end{array}$$ (19)

Incorporating the fault vector *m*(*k*) as an integral component of the system’s state representation, we construct an augmented state vector $\bar{x}\left(k\right)={\left[x\left(k\right)\;m\left(k\right)\right]}^{T}$. This expansion of the state space enables us to formulate a comprehensive augmented system that encapsulates both the original dynamics and the fault conditions. This approach not only enhances the descriptive power of our model but also facilitates the development of more sophisticated control strategies that can account for and adapt to fault-induced variations within the system.

$$\left\{ \begin{array}{c} \bar{E}\bar{x}\left(k+1\right)=\bar{A}\bar{x}\left(k\right)+\bar{B}u\left(k\right)\\ y\left(k\right)=\bar{C}\bar{x}\left(k\right) \end{array}\right.$$ (20)

where

$$\begin{array}{c} \bar{E}=[\begin{array}{cc} I_{n} & 0\\ 0 & 0 \end{array}],\bar{A}=[\begin{array}{c} A\;\;0\\ 0\;\;0 \end{array}],\bar{B}=[\begin{array}{c} B\\ 0 \end{array}],\bar{C}={\left[\begin{array}{c} C\\ N \end{array}\right]}^{T} \end{array}$$

In parallel with the development of the augmented system, we have successfully extended our observer design to accommodate this enhanced framework, as delineated by formula (4). The theoretical underpinnings that guarantee the feasibility of our observer are meticulously laid out in Theorem 1. While the constraints of this manuscript preclude a detailed repetition of the proof for the observer’s existence within this subsection, it is imperative to acknowledge the robust applicability of our observer design methodology.

### Observer design in situations of multiple modules being compromised considering uncertainties

In this subsection, we delve into a more complex scenario where multipoint faults are concurrently taken into account. To accurately capture the dynamics of the system under such conditions, we define the compromised system with a formulation that reflects the intricate interplay between these faults. The representation is given as follows:

$$\left\{ \begin{array}{c} x\left(k+1\right)=Ax\left(k\right)+Bu\left(k\right)+Mm\left(k\right)+E_{a}\omega_{a}\left(k\right)\\ y\left(k\right)=Cx\left(k\right)+Nm\left(k\right)+E_{s}\omega_{s}\left(k\right) \end{array}\right.$$ (21)

Within this analysis, the terms *ω*_*a*_(*k*) and *ω*_*s*_(*k*)represent unknown input vectors that encapsulate the inherent uncertainties present within the system. The matrices *E*_*a*_ and *E*_*s*_ are known constant coefficient matrices, each carefully dimensioned to align with the system’s structure. To adeptly incorporate the fault vector into our analytical framework, we introduce it as an additional state. This strategic maneuver allows us to construct the augmented state vector $\bar{x}\left(k\right)={\left[x\left(k\right)\;m\left(k\right)\right]}^{T}$, which embodies both the system’s state and the fault vector in a unified representation. With this enhanced state vector, we can articulate the following augmented system

$$\begin{array}{c} \left\{ \begin{array}{c} \bar{x}\left(k+1\right)=\bar{A}\bar{x}\left(k\right)+\bar{B}u\left(k\right)+\bar{E_{a}}\omega_{a}\left(k\right)+Gm^{d}\left(k\right)\\ y\left(k\right)=\bar{C}\bar{x}\left(k\right)+E_{s}\omega_{s}\left(k\right)\\ m^{d}\left(k\right)=m\left(k+1\right)-m\left(k\right) \end{array}\right. \end{array}$$ (22)

where

$$\begin{array}{c} \bar{A}=[\begin{array}{c} A\;\;M\\ 0\;\;I_{q} \end{array}],\bar{B}=[\begin{array}{c} B\\ 0 \end{array}],\bar{C}={\left[\begin{array}{c} C\\ N \end{array}\right]}^{T}\bar{E_{a}}={\left[\begin{array}{c} E_{a}\\ 0 \end{array}\right]}^{T}G={\left[\begin{array}{c} 0\\ I_{q} \end{array}\right]}^{T} \end{array}$$

Therefore, we can have

$$\begin{array}{c} \left\{ \begin{array}{cc} z\left(k+1\right) & =Rz\left(k\right)+Su\left(k\right)+\left(L_{1}+L_{2}\right)y\left(k\right)\\ \hat{\bar{x}}\left(k\right) & =z\left(k\right)+Ty\left(k\right) \end{array}\right. \end{array}$$ (23)

Therefore

$$\begin{array}{cc} e(k+1)= & \left(I-T\bar{C}\right)\bar{x}\left(k+1\right)-z\left(k+1\right)-TE_{s}\omega_{s}\left(k\right)\\ = & \left[\left(I-T\bar{C}\right)\bar{A}-L_{1}\bar{C}\right]e\left(k\right)+[\left(I-T\bar{C}\right)\bar{A}\\ & -L_{1}\bar{C}-R]z\left(k\right)+\left[\left(I-T\bar{C}\right)\bar{B}-S\right]u_{k}+\\ & \left[\left(\left(I-T\bar{C}\right)\bar{A}-L_{1}\bar{C}\right)T-L_{2}\right]y\left(k\right)\\ & +\left(I-T\bar{C}\right)\bar{E_{a}}\omega_{a}\left(k\right)+\left(I-T\bar{C}\right)Gm^{d}\left(k\right)\\ & -L_{1}E_{s}\omega_{s}\left(k\right)-TE_{s}\omega_{s}\left(k+1\right) \end{array}$$ (24)

If

$$\begin{array}{c} \left(I-T\bar{C}\right)\bar{E_{a}}=0 \end{array}$$ (25)

$$\begin{array}{c} \left(I-T\bar{C}\right)\bar{A}-L_{1}\bar{C}=R \end{array}$$ (26)

$$\begin{array}{c} \left(I-T\bar{C}\right)\bar{B}=S \end{array}$$ (27)

$$\begin{array}{c} RT=L_{2} \end{array}$$ (28)

We can have

$$\begin{array}{c} e\left(k+1\right)=Re\left(k\right)+\left(I-T\bar{C}\right)Gm^{d}\left(k\right)-L_{1}E_{s}\omega_{s}\left(k\right)-TE_{s}\omega_{s}\left(k+1\right) \end{array}$$ (29)

**Theorem 2.** Upon examination of the augmented system delineated in Eq 22, we postulate the existence of a robust observer, characterized by Eq 23. This observer is meticulously designed to ensure that the norm of the error vector *e*(*k*), when measured in the *l*_2_ space, is bounded by the inequality ${\left\|e\left(k\right)\right\|}_{l_{2}}\leq \sqrt{2}r{\left\|\gamma \left(k\right)\right\|}_{l_{2}}$.

The establishment of such a robust observer is contingent upon the fulfillment of a specific matrix inequality condition. This condition is met if one can ascertain the existence of a positive definite matrix *P* and a suitably dimensioned matrix *Q*, such that:

$$\begin{array}{c} {}[\begin{array}{cccc} -P+I_{\bar{n}} & * & * & *\\ 0_{l_{\gamma }\times \bar{n}} & -r^{2}I_{l_{\gamma }} & * & *\\ 0_{l_{\gamma }\times \bar{n}} & 0_{l_{\gamma }\times l_{\gamma }} & -r^{2}I_{l_{\gamma }} & *\\ PA_{1}-Q\bar{C} & PV_{1}-QV_{2} & P{\bar{V}}_{2} & -P \end{array}]<0 \end{array}$$ (30)

where $A_{1}=\left(I-T\bar{C}\right)\bar{A}$, *Q* = *PL*_1_, $V_{1}=\left[\left(I-T\bar{C}\right)G\;0_{\bar{n}\times l_{n}}\right]$, *V*_2_ = [0_*p*×*q*_ *E*_*s*_], and ${\bar{V}}_{2}=-TV_{2}$.

**Proof.** Considering that

$$\begin{array}{c} V\left(k\right)=e^{T}\left(k\right)Pe\left(k\right) \end{array}$$ (31)

We have

$$\begin{array}{cc} \Delta V(k)= & V(k+1)-V(k)\\ & ={\left[\begin{array}{c} e(k)\\ \gamma (k)\\ \gamma (k+1) \end{array}\right]}^{T}([\begin{array}{c} R\\ V_{1}-L_{1}V_{2}\\ {\bar{V}}_{2} \end{array}]P{\left[\begin{array}{c} R\\ V_{1}-L_{1}V_{2}\\ {\bar{V}}_{2} \end{array}\right]}^{T}\\ & +[\begin{array}{cc} -P & 0\\ 0 & 0 \end{array}])[\begin{array}{c} e(k)\\ \gamma (k)\\ \gamma (k+1) \end{array}] \end{array}$$ (32)

The system is asymptotically stable since *γ*(*k*) = 0.

And

$$\begin{array}{cc} \Gamma = & \sum_{k=0}^{\infty }(\Delta V\left(k\right)+e^{T}\left(k\right)e\left(k\right)-r^{2}\gamma^{T}\left(k\right)\gamma \left(k\right)\\ & -r^{2}\gamma^{T}\left(k+1\right)\gamma \left(k+1\right)) \end{array}$$ (33)

Therefore

$$\begin{array}{cc} \Gamma = & \sum_{k=0}^{\infty }{\left[\begin{array}{c} e(k)\\ \gamma (k)\\ \gamma (k+1) \end{array}\right]}^{T}[\left([\begin{array}{c} R\\ V_{1}-L_{1}V_{2}\\ {\bar{V}}_{2} \end{array}]P\right)P^{-1}\\ & \left(P[\begin{array}{c} R\\ V_{1}-L_{1}V_{2}\\ {\bar{V}}_{2} \end{array}]\right)+[\begin{array}{cc} -P+I & 0\\ 0 & -r^{2}I \end{array}]]\\ & \left[\begin{array}{c} e(k)\\ \gamma (k)\\ \gamma (k+1) \end{array}\right] \end{array}$$ (34)

Therefore

$$\begin{array}{l} \sum_{k=0}^{\infty }(e^{T}(k)e(k)-r^{2}\gamma^{T}(k)\gamma (k)\\ -r^{2}\gamma^{T}(k+1)\gamma (k+1))+V(\infty )-V(0)<0 \end{array}$$ (35)

We have

$$\begin{array}{c} \sum_{k=0}^{\infty }e^{T}\left(k\right)e\left(k\right)-2r^{2}\gamma^{T}\left(k\right)\gamma \left(k\right)<0 \end{array}$$ (36)

Proofed.

Drawing upon the proposed observer framework, we are equipped to deduce the observed data corresponding to the system’s variables, juxtaposed with the directly measured data. For the dispatcher, it is imperative to discern the congruence between the observed and measured data sets during periods of normal operation. This alignment serves as a benchmark for system integrity.

Conversely, it is equally vital for the dispatcher to detect and characterize any discrepancies between the two data sets that may emerge under compromised conditions. Such anomalies are indicative of potential system faults or malicious interventions, and their timely identification is crucial for implementing corrective measures.

To facilitate this dual analysis, robust statistical or algorithmic methods must be employed to systematically compare the observed and measured data.

## Identification scheme against fault based on dual source data

In this section, we delve into an in-depth analysis of a fault identification scheme tailored to effectively counteract faults by leveraging dual-source data. We propose a sophisticated relation-based detection network that is meticulously engineered to distill the inherent similarity within the dual-source data. This network is pivotal in unraveling the intricate patterns that may otherwise be obscured in the multidimensional data space.

- The conventional methodology for quantifying the similarity of dual-source data vectors via Euclidean distance presents a notable challenge; it presupposes a substantial degree of prior knowledge on the part of system defenders, which may not always be feasible in dynamic and complex operational environments. Recognizing this limitation, our research introduces an innovative approach that circumvents the need for extensive a priori understanding. In this paper, we present a novel framework comprising an embedding module coupled with a relation module, both of which are designed to autonomously extract the similarity from dual-source data. The embedding module is responsible for transforming the raw data into a lower-dimensional, information-rich representation. This process is achieved through advanced feature learning techniques that capture the underlying structure and relationships within the data without relying on manual feature engineering.
- Traditional machine learning methodologies typically necessitate the computation of distances within the feature space to facilitate identification tasks. This approach, inherently reliant on the geometry of the feature space, often requires a substantial volume of training data to achieve satisfactory performance. The demand for large-scale datasets poses significant challenges, including increased computational resources, potential overfitting, and the practical difficulties associated with data acquisition and labeling. In contrast, the research presented in this paper adopts a more nuanced and efficient strategy. We eschew the conventional paradigm of learning feature distances and instead propose a direct learning framework for discerning the relationships inherent in dual-source data. Our approach is predicated on the insight that understanding the interconnections between data sources can be more informative and less resource-intensive than traditional distance-based learning.

**Fig 1:**
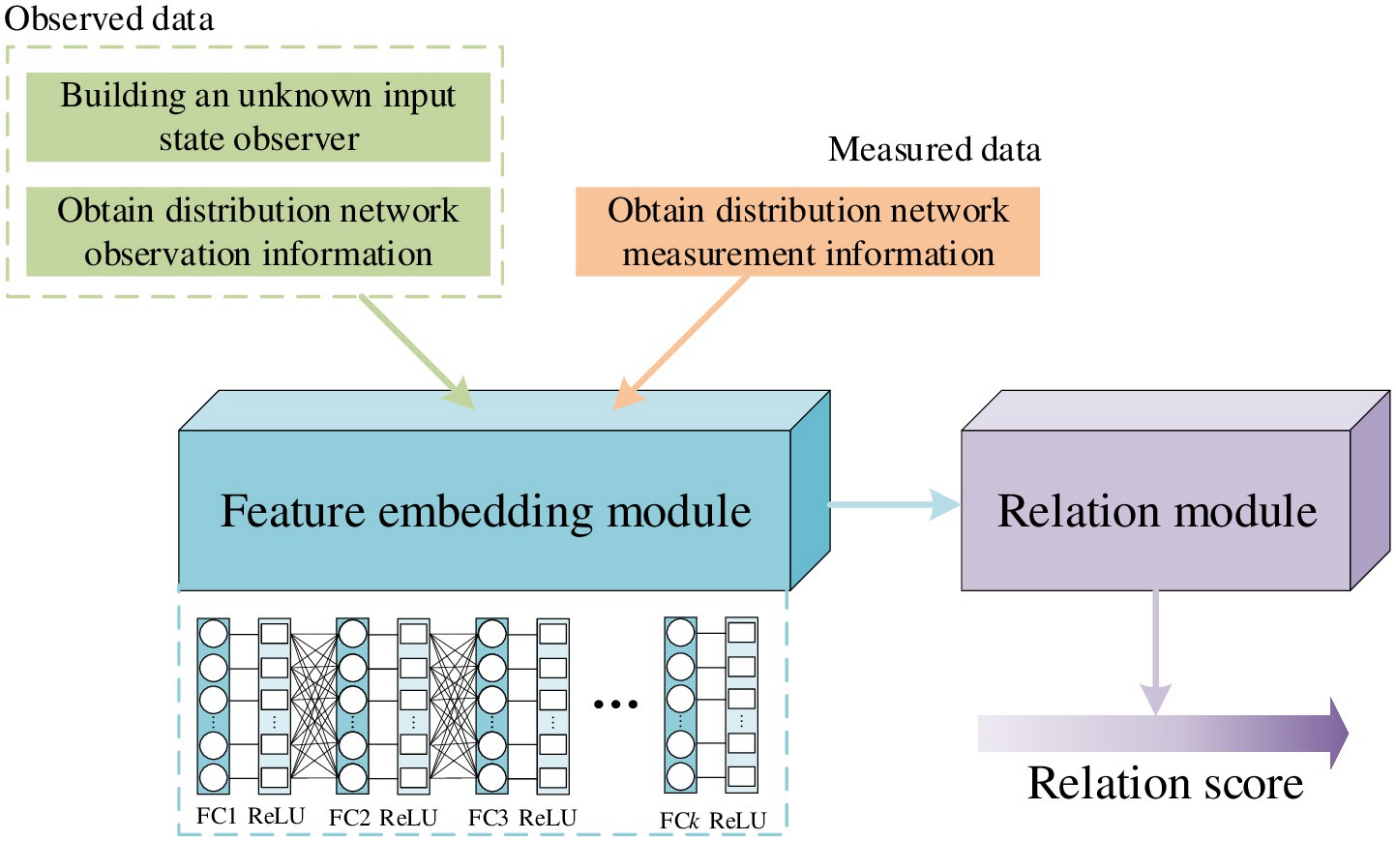
Schematic diagram of distribution network fault detection process.

As shown in Fig 1, we propose a twin network for detecting faults in distribution networks In the architecture of our identification network, we integrate a measured data set and an observed data set, alongside an embedding module and a relation module. The observed data set serves as a reliable reflection of the system’s current operational state, unadulterated by potential external manipulations. Conversely, the measured data set is a composite that includes both potentially compromised and normal data subsets, which necessitates careful scrutiny to ensure the integrity of the fault detection process.

Our fault detection network employs a comparative analysis between the observed data and the measured data to ascertain the presence of anomalies. This comparison is crucial as it allows us to distinguish between authentic operational data and data that may have been altered or tampered with.

To elaborate, when the comparative analysis involves data from the compromised subset, we typically observe a strong similarity in the relationship between the dual-source data. This strong similarity is indicative of a discrepancy that could suggest a fault or a cyber intrusion. On the other hand, when the comparison is drawn from the normal data subset, the relationship between the dual-source data exhibits weak similarity, which aligns with the expected behavior of a system free from faults or external interference.

The distinction between strong and weak similarity in the dual-source data relationship is pivotal for the accurate identification of faults. It is through this nuanced understanding of data relationships that our network can effectively discern and flag inconsistencies, thereby enhancing the reliability and security of the system it monitors.

In our dataset formulation, we represent each data vector as a time series encapsulating the dynamic behavior of the target variables. This time-dependent structure is critical for capturing the temporal correlations intrinsic to the system under study.

The core of our feature extraction process is an embedding module, ingeniously architected with fully connected layers and rectified linear units (ReLUs). This module employs a sophisticated nonlinear function, denoted by E, to distill the salient features from the raw sample data. This automated feature extraction paradigm, facilitated by the fully connected layers, offers a marked improvement over traditional manual techniques by substantially reducing the dependency on prior knowledge of fault characteristics. The FC layer operates on the principle of weighted connections, where each neuron in this layer is connected to all neurons in the previous layer. These connections are weighted, embodying the learned significance of each feature in the context of fault detection. The neurons in the FC layer combine these inputs linearly (using their respective weights), followed by the application of a non-linear activation function. By synthesizing this information, the FC layer can discern patterns indicative of normal operation or fault conditions within the distribution network. It effectively acts as a decision-making entity, where the learned weights reflect the importance of each feature in predicting the network’s status. The incorporation of ReLUs is a strategic choice aimed at enhancing the generalization capabilities of the embedding module, thereby enabling it to perform robustly across diverse operational scenarios. Upon processing by the embedding module, the feature vectors corresponding to the measured data and observed data are denoted by $F(d_{m})$ and $F(d_{o})$. These vectors encapsulate the distilled essence of the data, ready for subsequent analysis. In configuring the FC layer, one must consider the number of neurons, which directly correlates to the layer’s capacity to model complex relationships. A higher number of neurons allows for a more detailed representation but can also lead to overfitting and increased computational demands. Thus, the design of the FC layer requires a balance, ensuring it is sufficiently comprehensive to capture essential decision-making patterns while remaining computationally efficient and generalizable to unseen data. In the practical application of fault detection in distribution networks, it is necessary to fine tune the parameters of the FC layer based on the current operating scenario of the power grid, so as to maximize the detection of distribution network faults.

To address the perennial challenge of overfitting within the embedding module, we adopt a class prototype approach for each category of feature vectors. This innovative strategy involves the computation of representative prototypes for the feature vectors from both measured and observed datasets. The measured data feature vector prototype is represented as $P_{i}^{m}$, while the observed data feature vector prototype is denoted by $P_{i}^{o}$. These prototypes serve as archetypal points in the feature space, around which the corresponding class’s feature vectors are expected to cluster. This method not only mitigates the risk of overfitting but also provides a more intuitive understanding of the feature space structure, which is instrumental for the subsequent stages of fault detection analysis.

$$\begin{array}{c} P_{i}^{m}=\frac{1}{N_{i}^{m}}\sum_{j=1}^{N_{i}^{m}}F\left(d_{m}\right) \end{array}$$ (37)

$$\begin{array}{c} P_{i}^{o}=\frac{1}{N_{i}^{o}}\sum_{j=1}^{N_{i}^{o}}F\left(d_{o}\right) \end{array}$$ (38)

Within our analytical framework, let $N_{i}^{m}$ and $N_{i}^{o}$ denote the number of samples for class *i* within the measured and observed data feature vectors, respectively. To construct the class feature vector, denoted as $C(P_{i}^{m},P_{i}^{o})$, we employ a methodical approach by concatenating the class prototypes The relation module, an integral component of our system, leverages a nonlinear relation function R to discern the degree of similarity between these concatenated class feature vectors. The similarity metric, S is thus formulated as:

$$\begin{array}{c} S=R(C\left(P_{i}^{m},P_{i}^{o}\right)) \end{array}$$ (39)

Mean square error (MSE) is used to train the proposed identification loss *L*_*m*_.

$$\begin{array}{c} L_{m}=\{\begin{array}{c} \sum \sum {\left(S-1\right)}^{2},l_{m}=l_{o}\\ \sum \sum {\left(S-0\right)}^{2},l_{m}\neq l_{o} \end{array} \end{array}$$ (45)

In our analysis, we denote *l*_*m*_ and *l*_*o*_ as the labels corresponding to the measured and observed data, respectively. These labels are paramount as they represent the ground truth against which the integrity of the data is assessed.

The crux of our fault detection algorithm hinges on the comparison of these labels. In the event of a discrepancy between the measured data and the observed baseline, indicated by *l*_*m*_ ≠ *l*_*o*_ and S is closed to 0.

## Case study

In this section, we present a series of simulations designed to rigorously evaluate the efficacy of our proposed observer and the associated fault detection network for monitoring system variables.

### Performance of the observer for the compromised system

In the test system, let

$$\begin{array}{c} A=[\begin{array}{cccc} 0.9988 & 0.0007 & 0.0006 & -0.0037\\ 0.0014 & 0.98 & -0.0012 & -0.0206\\ 0.001 & 0.0037 & 1.0467 & 9.5584\\ 0 & 0 & 0.0101 & 1.0234 \end{array}] \end{array}$$

$$\begin{array}{c} B=[\begin{array}{cc} 0.0052 & 0.0012\\ 0.0315 & -0.0755\\ -0.0582 & 0.0454\\ -0.0003 & 0.0002 \end{array}],C=[\begin{array}{cccc} 0.45 & 0.32 & 0.12 & 0.11\\ 0.38 & 0.42 & 0.13 & 0.07\\ 0.27 & 0.31 & 0.33 & 0.09\\ 0.07 & 0.13 & 0.43 & 0.37 \end{array}] \end{array}$$

where

$$\begin{array}{c} m_{1}\left(k\right)=\{\begin{array}{c} 0,\;k<60\\ 0.05(k-60),\;60\leq k<100\\ 2,\;k\geq 100 \end{array} \end{array}$$ (41)

$$\begin{array}{c} m_{2}\left(k\right)=\{\begin{array}{c} 0,\;k<60\\ 1,\;k\geq 60 \end{array} \end{array}$$ (42)

In this section, we commence by delineating the efficacy of our observer’s fault handling capabilities. We focus on the fault target variable $x_{P}^{p}$, which serves as a key indicator within our system’s diagnostic framework.

**Fig 2:**
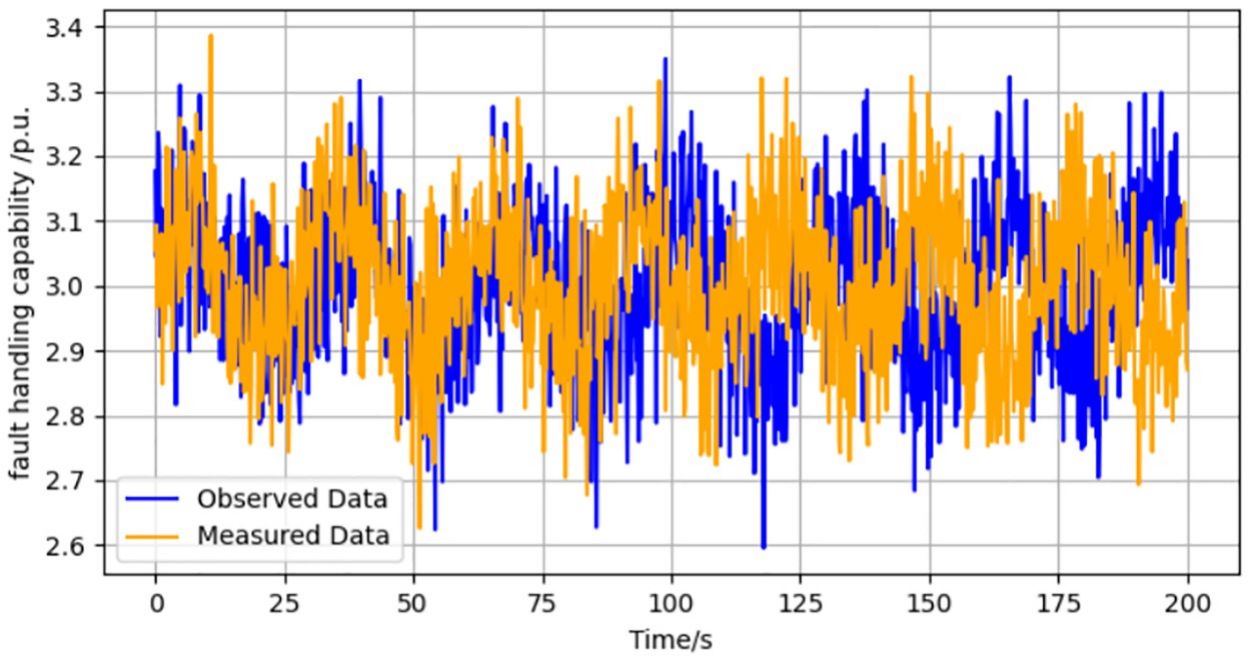
Dual source data of variable $x_{p}^{P}$ under faults on fault handling capabilities.

Employing the methodology delineated in the paper, we acquire the observed data for the variable $x_{P}^{P}$. The fidelity of the observer is then assessed through a series of simulations, the outcomes of which are visually encapsulated in Fig 2. These simulations provide a dual-source data comparison, juxtaposing observed and actual measurements.

**Fig 3:**
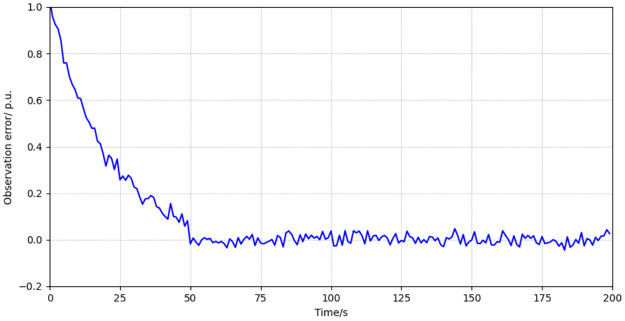
Observation error of variable $x_{p}^{P}$ under faults on fault handling capabilitie.

Furthermore, the discrepancies between the observed and measured data are quantitatively depicted in Fig 3 showcasing the observation error. Our analysis reveals that when the fault magnitude is static, the observer exhibits a commendable capacity to track the measured data with high precision. Conversely, in scenarios where the fault magnitude is dynamic, a discernible observation error emerges. This error is attributable to the dynamic nature of the fault, which introduces a variable disturbance akin to a moving target for the observer.

The magnitude and behavior of the observation error are not merely artifacts; rather, they are instrumental in the operationalization of our fault detection network. The disparity between the observed and measured data forms a critical metric, serving as a cornerstone for the network’s algorithms to ascertain the integrity of the system. It is this nuanced interplay between observed discrepancies and fault detection that underpins the robustness of our proposed diagnostic approach.

Subsequently, we rigorously evaluate the observer’s performance in the context of faults affecting the output power decision-making process. The focal point of this assessment is the fault target variable $y_{C}^{p}$, which is indispensable for ensuring the reliability and accuracy of power output decisions.

Leveraging the methodology articulated in the paper, we derive the observed data for the state variable $x_{C}^{p}$. This process is underpinned by a robust analytical framework designed to capture the nuances of the observer’s operation under fault conditions.

**Fig 4:**
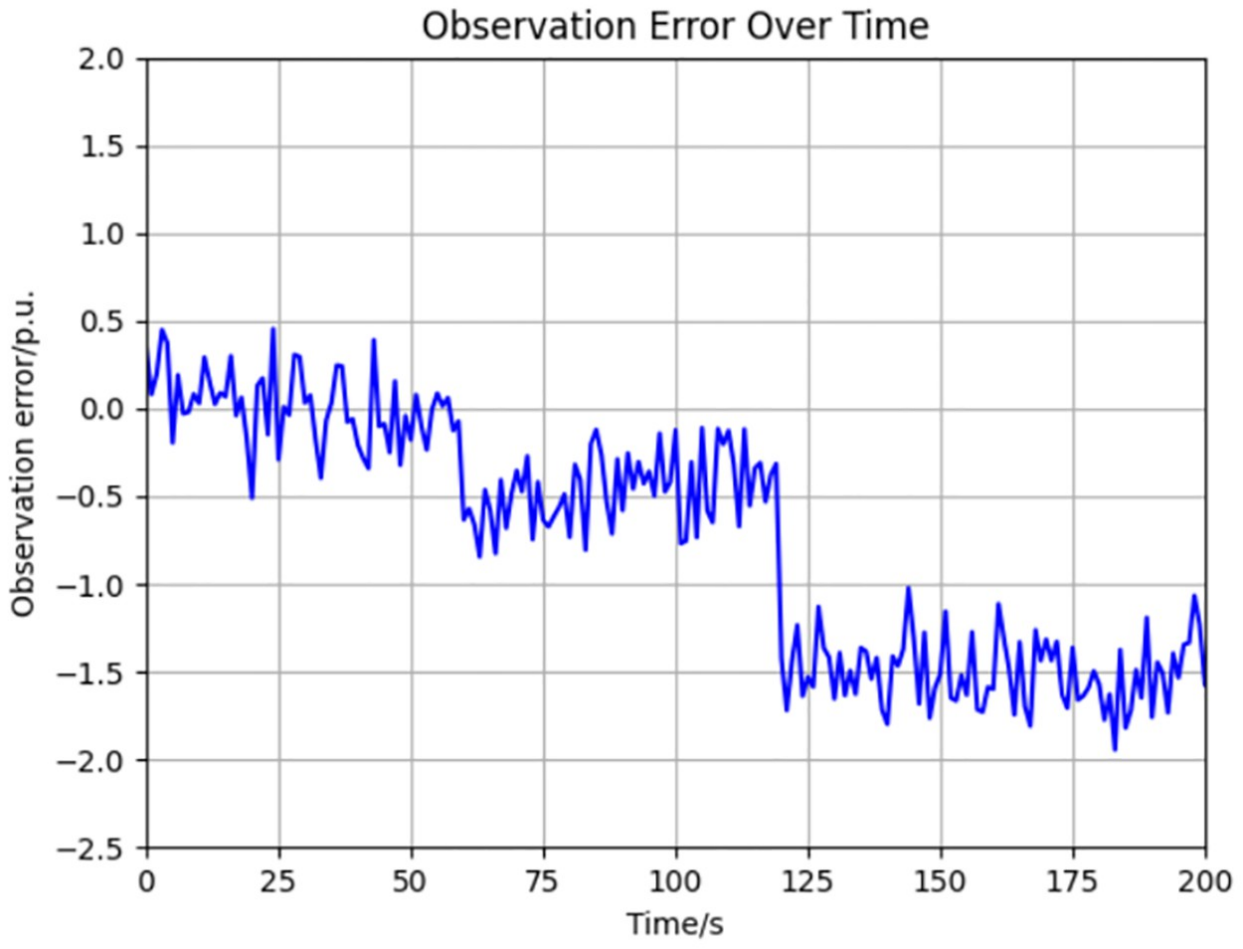
Observation error of variable $x_{p}^{C}$ under faults on fault handling cost.

The results of our simulations, which are pivotal to our investigation, are depicted in Fig 4. These figures are not merely illustrative but are also analytical tools that provide insight into the observer’s performance metrics and fault tolerance capabilities.

It is imperative to note that the simulation results extend beyond a mere presentation of data; they encapsulate the observer’s ability to maintain the integrity of the decision-making process in the face of perturbations. The implications of these findings are profound, informing the development of more resilient and secure power system infrastructures.

To elucidate the robustness of the proposed observer when confronted with the simultaneous compromise of multiple modules, we conduct a detailed analysis of simulation results under these multifaceted fault conditions. This analysis is crucial for understanding the observer’s performance in a realistic scenario where multiple variables may be subjected to adversarial interventions.

**Fig 5:**
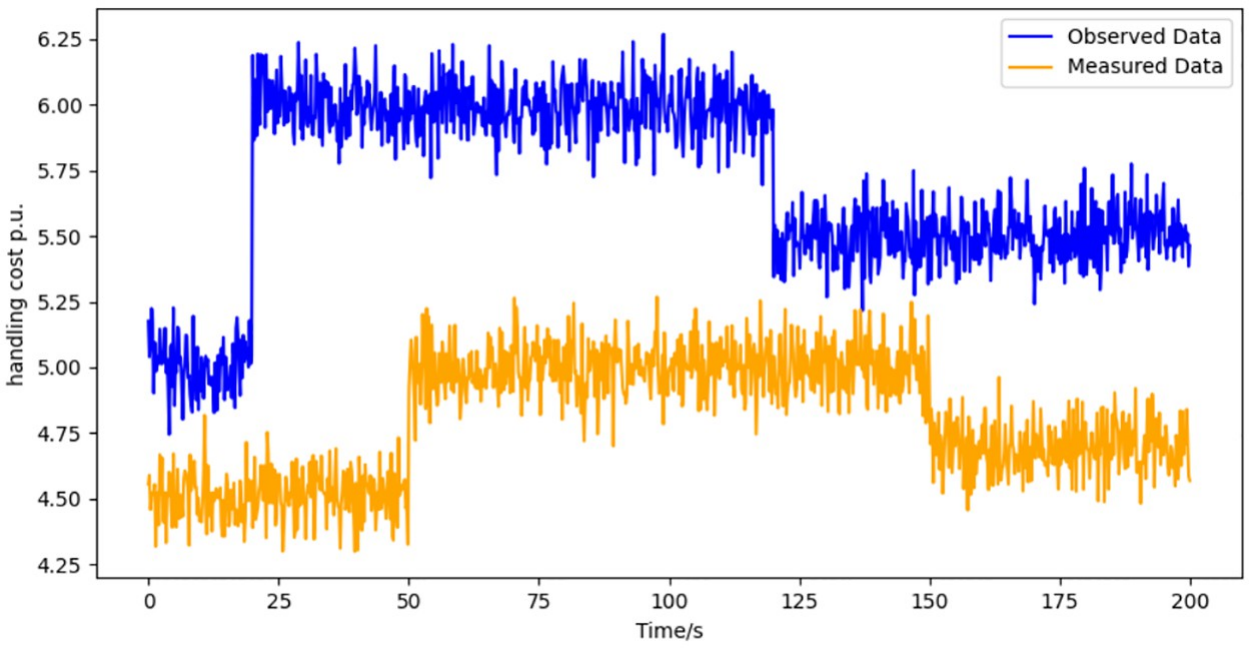
Dual source data of variable $x_{h}^{C}$ under faults on multiple modules.

Employing the proposed methodology, we obtain the observed data for the compromised variables. The fidelity of our observer is then rigorously assessed through simulations, the results of which are presented in Fig 5. These figures are not merely visual aids but serve as critical evidence of the observer’s diagnostic capabilities.

A careful examination of the simulation outcomes reveals pronounced discrepancies between the measured data and the observed data. This discrepancy is indicative of the complex interplay between the fault magnitude and other system perturbations, such as noise, disturbances, and delays. Such findings underscore the imperative for a sophisticated fault detection scheme capable of discerning whether the system’s integrity has been undermined.

Accordingly, the necessity for an effective fault detection mechanism becomes apparent, one that can reliably ascertain the presence of a compromise within the system. This mechanism is integral to the maintenance of system reliability and security, particularly in the presence of faults that could otherwise go undetected.

### Performance of the observer for the relation-based fault detection scheme

In this subsection, we undertake a comprehensive evaluation of the performance of the proposed fault detection scheme, which is a cornerstone of our research. The architecture of the embedding module within this scheme incorporates three fully connected layers, each followed by rectified linear units (ReLUs) to introduce non-linearity and enhance feature learning capabilities.

For the training of the relation network, we judiciously select a batch size of 20. This choice balances computational efficiency with the network’s ability to generalize from the training data. In compiling the measured data set, we draw upon a historical database to amass 500 samples representative of normal system behavior and an additional 500 samples indicative of compromised system states. This balanced approach ensures that the fault detection scheme is exposed to an array of conditions reflective of the system’s operational spectrum.

In parallel, the observer generates a set of 1000 observed data points, employing the methodological framework proposed. These observed data serve as the testbed for our fault detection scheme, providing a robust dataset against which the scheme’s efficacy is measured.

To ascertain the fault detection scheme’s performance with precision, we employ a suite of indexes as follows.

1) MA:

$$\begin{array}{c} MA=\frac{TP+TN}{TP+TN+FP+FN} \end{array}$$ (43)

2) MD:

$$\begin{array}{c} MD=\frac{TP}{TP+FN} \end{array}$$ (44)

3) MS:

$$\begin{array}{c} MS=\frac{TP}{TP+FP} \end{array}$$ (45)

4) MI:

$$\begin{array}{c} MI=\frac{TN}{TN+FP} \end{array}$$ (46)

5) MF:

$$\begin{array}{c} MF\left(T\right)=\frac{(1+T^{2})\cdot MD\cdot MS}{T^{2}\cdot MD+MS} \end{array}$$ (47)

where TP represents the number of samples correctly predicted as positive class, TN represents the number of samples correctly predicted as negative class, FP represents the number of negative class samples incorrectly predicted as positive class, FN represents the number of positive class samples incorrectly predicted as negative class. MA represents accuracy, MD represents precision, MS represents recall, MI represents false detection rate, and MF represents a comprehensive performance indicator that can comprehensively consider the performance of the model in terms of both miss detection rate and false detection rate.

**Fig 6:**
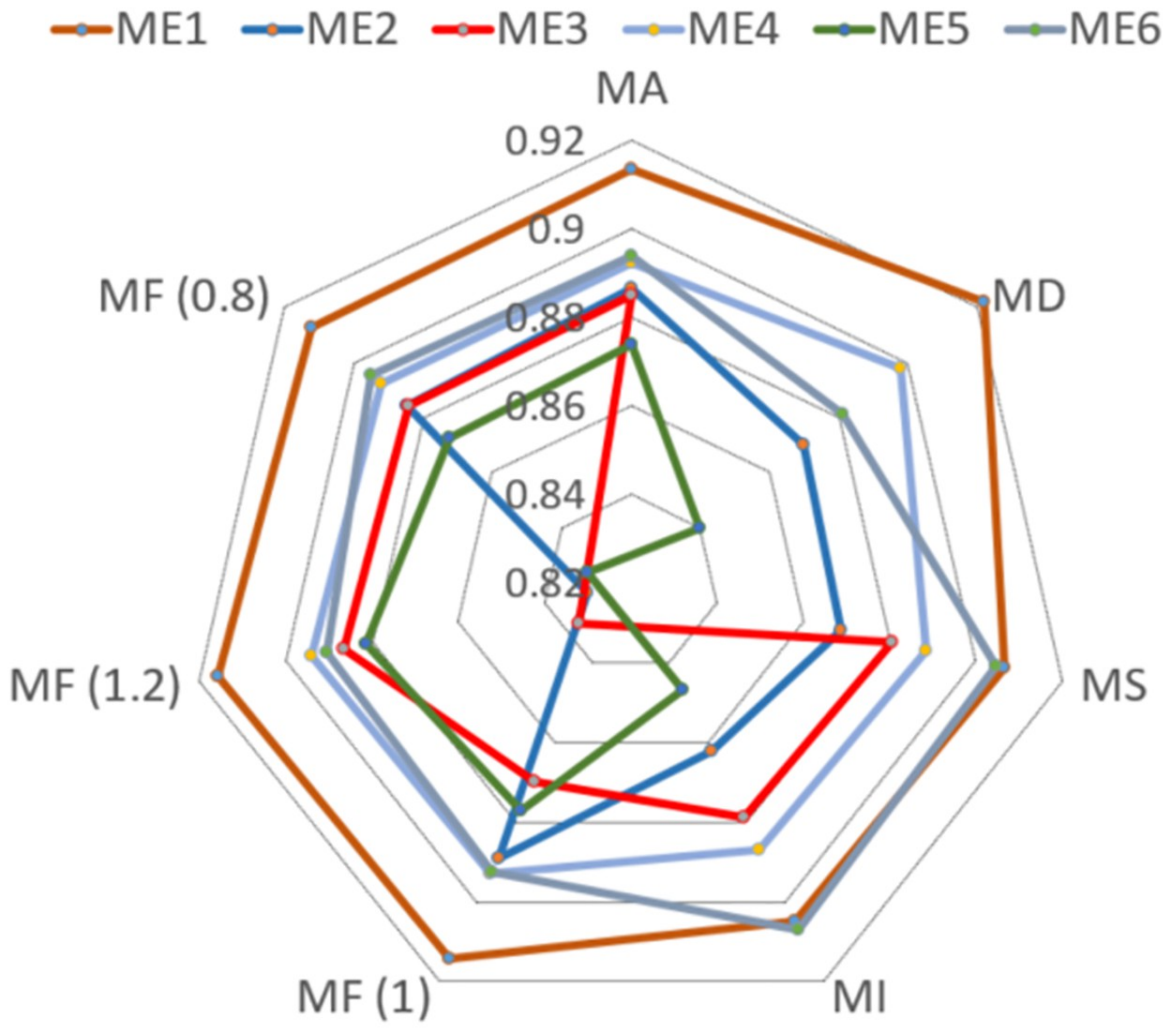
Performance of different fault detection method.

To comprehensively evaluate the efficacy of our proposed relation-based fault detection scheme, we have rigorously compared it against a suite of five alternative methods. These methods, each with its distinct approach to fault detection, are enumerated as follows:

ME1: Our novel relation-based fault detection scheme, which stands at the forefront of our research. ME2: A related fault detection scheme utilizing a relation network, yet lacking the prototype module, to discern the value added by this component [22]. ME3: A fault detection scheme employing a multilayer perceptron, representing a standard in neural network applications [23]. ME4: A scheme that applies signal forecasting methods, offering a perspective on the utility of time-series predictive analysis in fault detection [24]. ME5: A support vector machine-based fault detection scheme, capitalizing on the strengths of SVMs in pattern recognition [25]. ME6: A fault detection scheme that incorporates a clustering artificial bee colony algorithm, reflecting the innovative use of swarm intelligence in anomaly detection [26]. Our simulation results, as depicted in Fig 6, clearly demonstrate that the proposed fault detection scheme (ME1) outperforms its counterparts across all algorithmic evaluation indices. This superior performance is attributable to the scheme’s strategic focus on exploiting the discrepancies between normal and compromised data. In contrast, the other fault detection schemes primarily emphasize feature extraction.

The distinctive advantage of the proposed scheme arises from its ability to discern the subtle variances that distinguish authentic data from manipulated data. This is in stark contrast to other schemes, which may inadvertently learn commonalities between normal and compromised data, thereby diminishing their detection capabilities.

In pursuit of a comprehensive understanding of the stability and robustness of our proposed fault detection scheme, we have conducted an in-depth analysis of its performance across varying training set sizes. Specifically, we have incrementally adjusted the proportion of the data used for training by 5% intervals, ranging from 40% to 80% of the total dataset.

**Fig 7:**
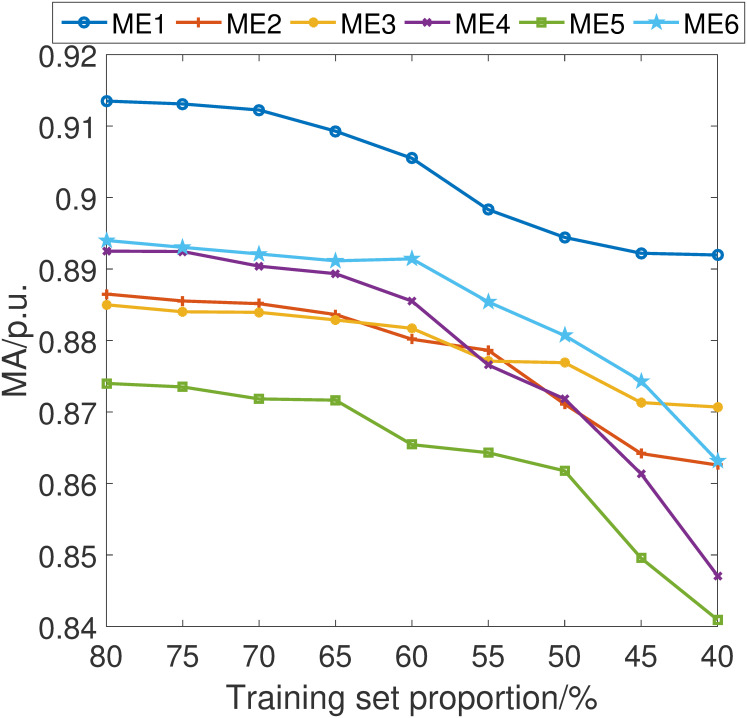
MA index considering different training set proportion.

**Fig 8:**
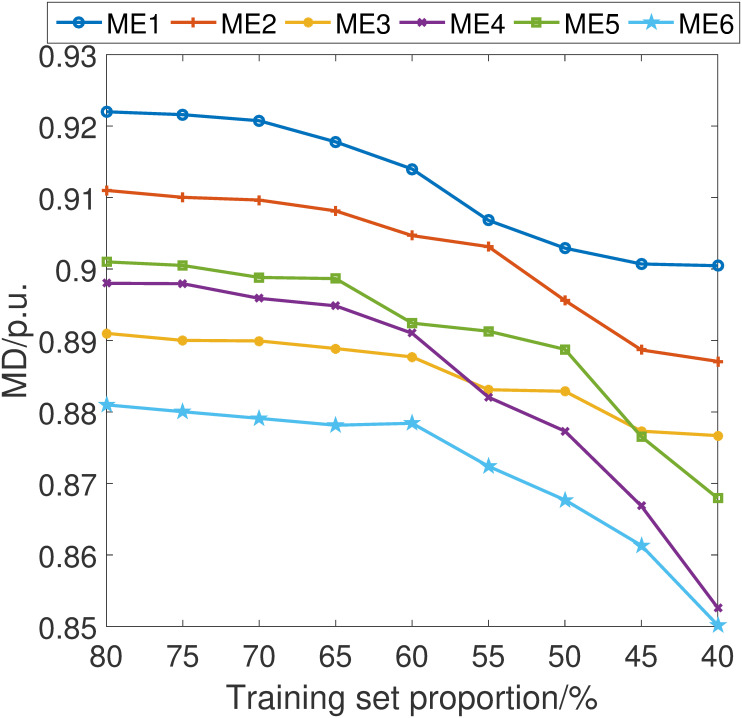
MD index considering different training set proportion.

**Fig 9:**
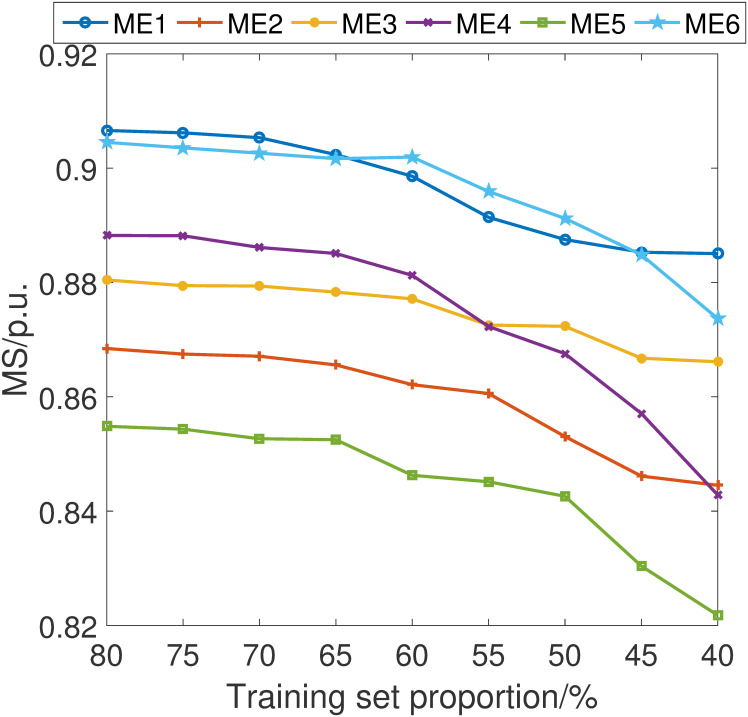
MS index considering different training set proportion.

**Fig 10:**
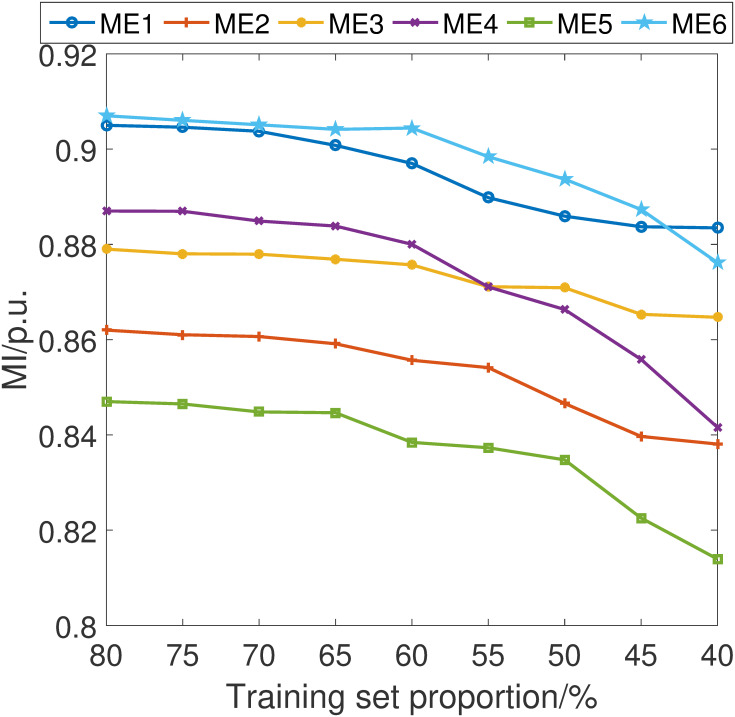
MI index considering different training set proportion.

The simulations are shown in Figs 7–10. It can be learned that while there is an expected decline in the performance of the proposed fault detection scheme as the training sample size decreases, it nonetheless maintains a position within the upper echelon of performance metrics. This observation holds true even when compared with alternative schemes, some of which may occasionally surpass our method at certain training sample sizes. Crucially, these results confirm that our fault detection scheme consistently delivers an excellent detection capability within the sample size parameters explored in this study.

The resilience of the proposed scheme, as evidenced by these simulations, underscores its robustness and affirms its effectiveness in fault detection, even under constraints of limited data availability. This finding is particularly significant for practical applications where data may be scarce or costly to acquire, further attesting to the versatility and practicality of our approach.

In light of the practical scenario where compromised data samples may be sparse, we have undertaken a thorough examination of the proposed fault detection scheme’s reliability and stability across varying ratios of positive (compromised) to negative (normal) samples. We have methodically evaluated the scheme at the following ratios: 1:1, 1:2, 1:5, and 1:10, thereby encompassing a broad spectrum of imbalanced datasets.

**Fig 11:**
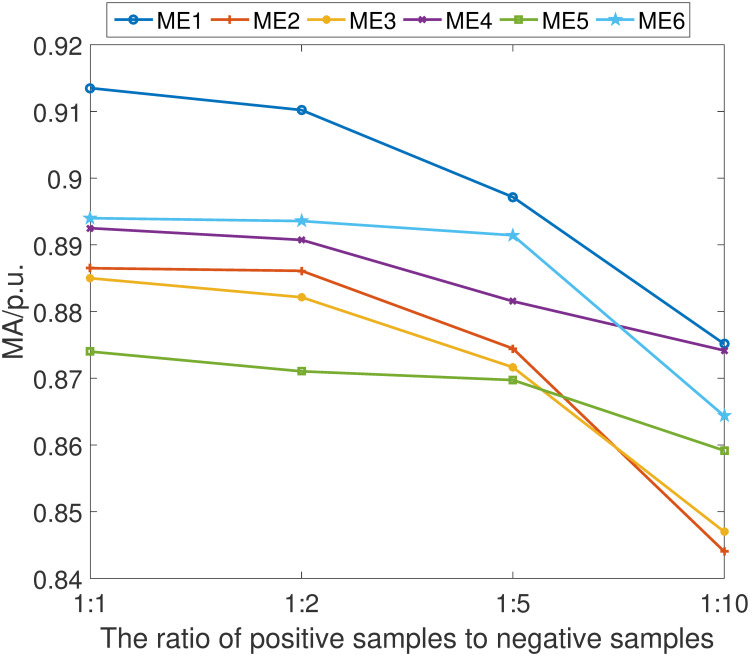
MA index under different ratio of positive samples.

**Fig 12:**
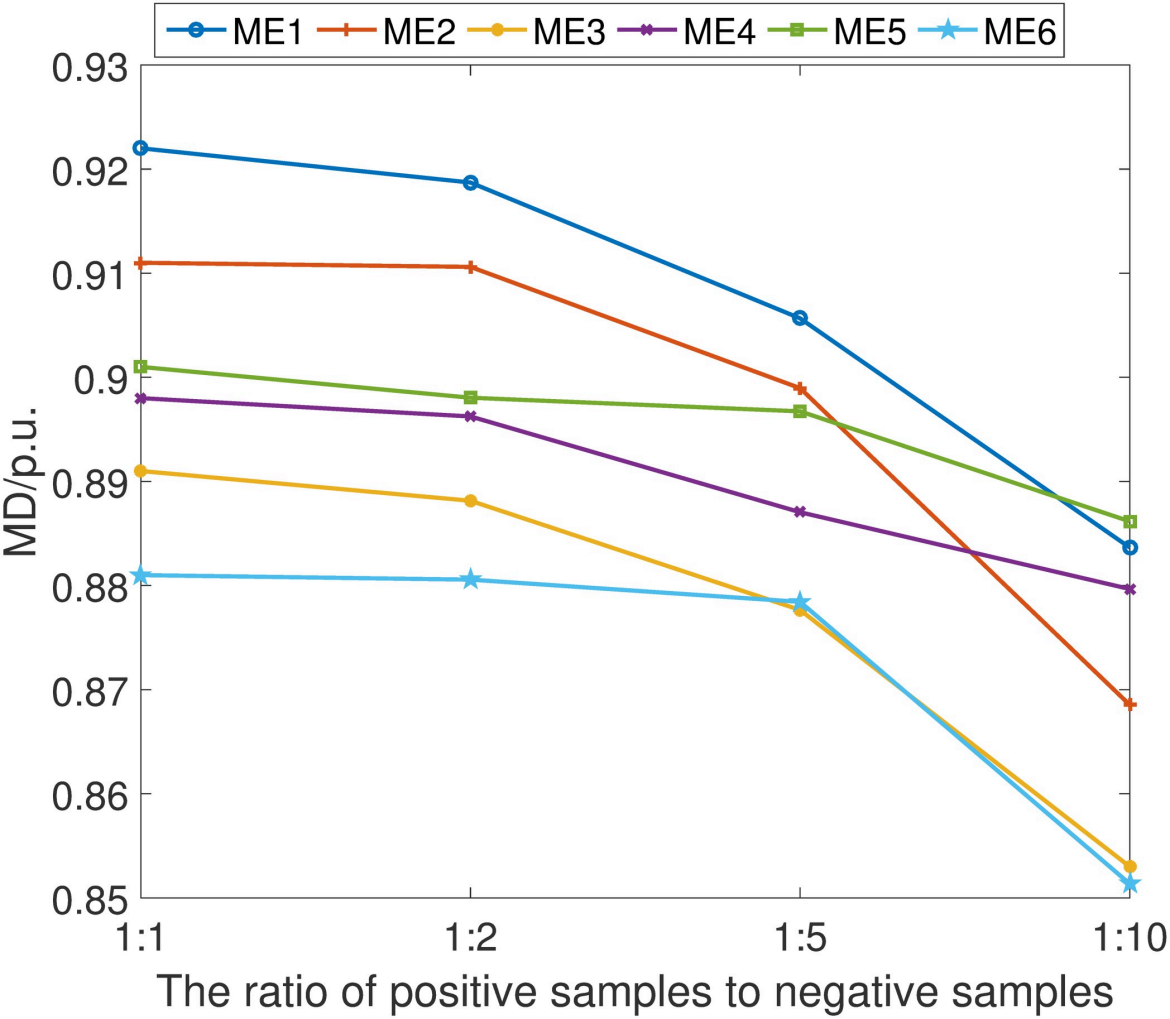
MD index under different ratio of positive samples.

**Fig 13:**
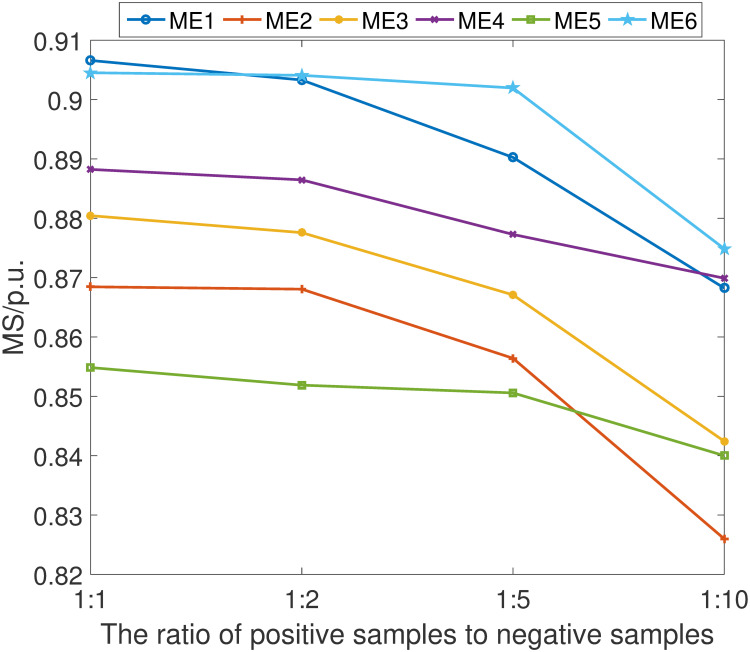
MS index under different ratio of positive samples.

**Fig 14:**
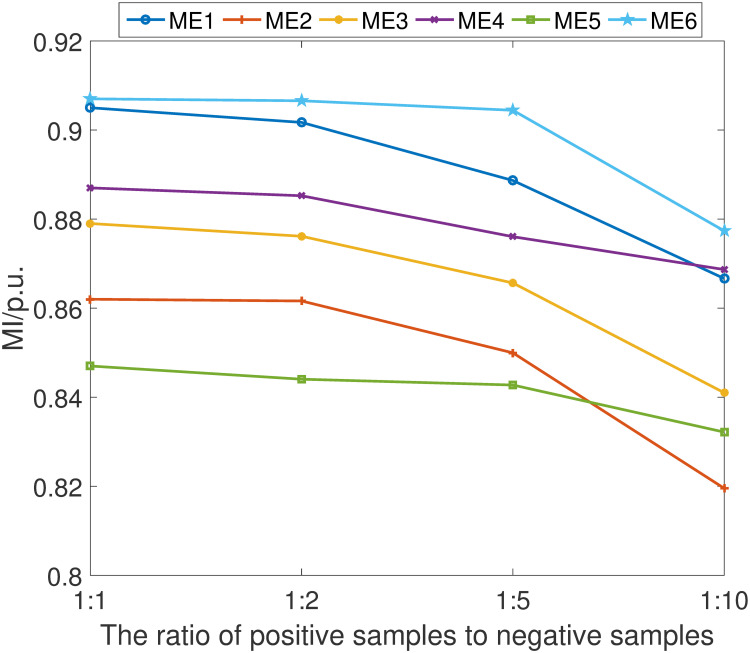
MI index under different ratio of positive samples.

The empirical results are shown in Figs 11–14. It can be learned that while there is a discernible decrement in the scheme’s performance as the proportion of positive samples diminishes relative to negative samples, the scheme’s overall efficacy remains comparatively advantageous against other detection methods. This performance degradation is primarily attributed to the network’s constrained ability to adequately learn and distinguish the characteristics of positive samples when they are underrepresented.

Despite this challenge, it is noteworthy that the proposed fault detection scheme continues to demonstrate a commendable level of fault identification accuracy. This resilience is a testament to the robust design of our detection network, which has been engineered to cope with the inherent difficulties posed by sample imbalance. Furthermore, the findings elucidate the critical need for a detection system that can maintain high performance levels even when faced with the realistic limitations of data availability, thus reinforcing the practical applicability and superiority of our proposed scheme in real-world settings.

## Conclusion

In this study, we address the critical issue of fault management within the distributed controllers of active distribution networks. To this end, we have meticulously developed observers capable of tracking anomalous data indicative of system faults. These observers have been intricately designed to accommodate a variety of fault targets, ensuring a comprehensive monitoring framework. Building upon the data procured by these observers, we have introduced an innovative relation-based fault detection scheme. This scheme leverages the interdependencies between observed data and actual measurements to pinpoint discrepancies that may signal the presence of faults. The simulation results robustly validate the efficacy of our observers and the relation-based fault detection scheme.
